# Antimicrobial Therapy in Pediatric Sepsis: What Is the Best Strategy?

**DOI:** 10.3389/fped.2022.830276

**Published:** 2022-02-15

**Authors:** Luciana Becker Mau, Vera Bain

**Affiliations:** ^1^Department of Hospital Epidemiology and Infection Control, Hospital Municipal Infantil Menino Jesus, São Paulo, Brazil; ^2^Pediatric Infectious Diseases Unit, Hospital das Clínicas, Instituto da Criança, Universidade de São Paulo (HC-FMUSP), São Paulo, Brazil

**Keywords:** pediatric sepsis, antibiotic, antimicrobial stewardship, vaccination, education

## Abstract

Pediatric sepsis is a relevant cause of morbidity and mortality in this age group. Children are affected differently in high and low-income countries. Antibiotics are crucial for the treatment of sepsis, but indiscriminate use can increase resistance worldwide. The choice of a correct empiric therapy takes into consideration the site of infection, local epidemiology, host comorbidities and recent antibiotic exposure. Antibiotics should be administered in the first hour for patients with septic shock, and always intravenously or *via* intraosseous access. Culture results and clinical improvement will guide de-escalation and length of treatment. New diagnostic methods can help improve the prescription of adequate treatment. Prevention of sepsis includes vaccination and prevention of healthcare-associated infections. More research and education for awareness of sepsis is needed to improve care.

## Introduction

### Why Talk About Sepsis?

Severe sepsis and septic shock are responsible for 10–25% of Pediatric Intensive Care Unit (PICU) admissions worldwide (1, 2). As many as 30–50% of patients come from other hospital wards whereas the rest are admitted directly from the Emergency Department. Pediatric chronic conditions are a relevant risk factor, with 77% of patients having at least one comorbidity (1).

Sepsis morbidity and mortality affect children differently around the world (1–3). Although international data shows a mortality of 25% and the presence of long-term sequelae in 20% of survivors, it is known that children living in low- and middle-income countries face the highest burden (1). Factors that contribute to this disparity are sanitary conditions, level of maternal education, vaccine coverage, access to the health care system, having bundles and protocols for sepsis recognition and treatment and local rates of antimicrobial resistance and healthcare-associated infections (3–6).

In Brazil, the estimated incidence of severe sepsis and septic shock is 74.6 cases per 100,000 pediatric population, resulting in 42,374 cases per year and 8,305 deaths (2).

## Antibiotics: Step by Step

### Antimicrobial Use: Importance and Risks

Antibiotics are the most prescribed drugs in pediatrics (7). Since its discovery more than 90 years ago, antibiotics have been widely used for the treatment of infections. Every launch of a new class of antibiotics is accompanied by a description of new mechanisms of bacterial resistance to antimicrobials (8). Unfortunately, the process of development of new drugs is long and difficult, and the rate of resistance is constantly increasing (7–9). It is estimated that, without rational use of antibiotics, in 2050 over 10 million people will die every year due to drug resistant infections (10).

Antibiotics are crucial for the treatment of sepsis. Timely prescription of antimicrobials is one of the main goals to prevent mortality (11). Especially for patients with shock and end-organ disfunction, the ideal moment for receiving antibiotics is within the first hour of health assistance (4, 5, 12, 13). However, the indiscriminate use of antibiotics can lead to increased resistance, risk of *Clostridioides difficile* infection, as well as drug related toxicity (acute kidney injury, diarrhea, cytopenia, skin rash and anaphylaxis) (7, 9).

### Choosing the Best Drug

Empiric therapy should be carefully prescribed, as it will be the first therapy the patient will receive when presenting with sepsis and septic shock, and selection of an appropriate drug can save lives. We suggest evaluating these principles when choosing the empiric therapy: identification of likely agent according to infection site and knowing the local susceptibility profile, evaluating the risk of having a bacterial infection, the severity of the disease, the host comorbidities and recent admissions and usage of antibiotics (14, 15) (Table 1).

- Determining the site of infection is important to the choice of correct drug. Doses of antibiotics tend to be higher for central nervous system infections, for example. Also, resistance of pathogens also varies according to infection site. When choosing a drug to treat pneumonia in children, it is mandatory to know the epidemiological profile of *Streptococcus pneumoniae* resistance to beta lactams, for instance. Local guidelines are available in most countries. For healthcare-associated infections, information can be obtained with Infection Control Committees within each hospital (14, 15).
- Some clues can be used to evaluate the risk of really having a bacterial infection. Most illnesses in children are viral, and the patient tends to improve without treatment by day 3 to 5. The child is usually well between episodes of fever and laboratory results tend to be normal, with mildly elevated biomarkers. Not prescribing antibiotics for probable viral infections can save up to 70% of antimicrobial prescriptions (14).
- The severity of the patient is also important to define the therapy. In patients with septic shock, there is no possibility of waiting for culture results to choose the best drug. Broad-spectrum antibiotics are indicated for unstable patients, as there will be no time for escalation of therapy if response to the first drug is inadequate. Bactericidal drugs are preferable over bacteriostatic ones (11, 14, 15).
- Host comorbidities and recent admissions and use of antimicrobials will also shape the decision. Oncologic patients recently submitted to chemotherapy have a higher risk of febrile neutropenia and *Pseudomonas aeruginosa* infections. Having an indwelling medical device increases the risk of infection, with *Staphylococcus aureus* being an important agent in patients with central venous catheters (CVC). Recent use of antibiotics can increase risk of resistance, varying between 30 and 90 days according do different literature (14, 16–18). Patients with CVC, recently exposed to abdominal surgery, immunosuppressed or neutropenic, receiving total parenteral nutrition, with prolonged intensive care unit (ICU) stay and prolonged exposure to broad spectrum antibiotics are at higher risk of fungal infections (14).
- After considering all these conditions we can also evaluate costs, drug availability, risk of toxicity and side effects (7).

**Table 1:** Most common antibiotics use in clinical practice for community acquired infection.

**Clinical syndrome**	**Suggestion of treatment**
Sepsis without source	Ceftriaxone 100 mg/kg/day
Central nervous system	Ceftriaxone 100 mg/kg/day. Add Vancomycin divided q6h when *Streptococcus pneumoniae* resistance is relevant
Neutropenic fever in cancer patients during chemotherapy	Cefepime 150 mg/kg/day divided q8h or Piperacillin-Tazobactam 300 mg/kg/day divided q6h to cover *Pseudomonas aeruginosa*
Patients with central venous lines	Add Vancomycin 40 mg/kg/day divided q6h to cover *Staphylococcus aureus*
Abdominal infection	Ceftriaxone 50 mg/kg/day + Metronidazole 40 mg/mg/day divided q6h
Biliary Involvement	Ceftriaxone 50 mg/kg/day + Ampicillin 200 mg/kg/day divided q6h
Toxic shock	Oxacillin* 200 mg/kg/day divided q6h or methicillin + Clindamycin 40 mg/kg/day divided q6-8h

*In all cases: attention to prior colonization, prior antibiotic use and hospitalization to access the risk of multidrug resistant agent INFECCION (vancomycin-resistant Enterococcus, methicillin-resistant Staphylococcus aureus and carbapenem-resistant Enterobacteriaceae)*.

### Specific Considerations on Antibiotic Selection and Drug Resistance

NICE guidelines indicate that the best drug to treat community acquired sepsis and septic shock is ceftriaxone (19). With recent increase in pneumococcal resistance to ceftriaxone in central nervous system infections, it is also recommended to include vancomycin in empiric therapy of patients with meningitis (20, 21).

Some countries have a high frequency of community acquired infections caused by methicillin-resistant *Staphylococcus aureus* (MRSA). In these settings, prescription of vancomycin, linezolid, daptomycin or ceftaroline is recommended for unstable patients with suspected staphylococcal infections, such as skin and soft tissue infections (15). Clindamycin is also relevant as an adjunctive therapy for the treatment of toxic shock, owing to its activity against staphylococcal and streptococcal toxins (22).

Abdominal infections are frequently polymicrobial. Antibiotic coverage for gram negative bacteria and anaerobes is indicated for all cases. Ampicillin or other drugs active against *Enterococcus* should be added to the empiric therapy when the biliary tract is the source of infection or after surgical manipulation of the biliary tract (23).

Recent exposure to antibiotics, recent hospital admission and colonization with multidrug-resistant pathogens will shape the choice of empiric therapy. The Surviving Sepsis Campaign 2020 Guidelines suggest considering these factors when prescribing antibiotics but does not define the exact time of recent exposure to antibiotics (11). A Spanish study found a higher rate of penicillin resistant *Staphylococcus aureus* in patients with penicillin prescription in the last 4 years (16). A Canadian study showed that risk of pneumococcal resistance is increased after a course of penicillin, cephalosporins, quinolones and macrolides. The rate of resistance returns to basal after 90 days of exposure for the first three classes of antimicrobials but remains higher for a longer period for macrolides (17). A multicentric study in Europe evaluated the risk of colonization with extended spectrum beta lactamase (ESBL) gram-negative bacteria and found that antibiotic prescription during admission, especially cephalosporins, was the main risk factor for acquiring new colonization (18).

Colonization with resistant bacteria such as carbapenem-resistant *Enterobacteriaceae* and vancomycin-resistant *Enterococci* increases the risk of infection with the same agent. A study identified carbapenem-resistant *Klebsiella pneumoniae* (CR-KP) infections in 37% of 62 patients with rectal colonization. The risk factors for developing infection among carriers were being admitted to the ICU, receiving a CVC and being in a coma. Interestingly, no pediatric patients developed infections (24). Giannella et al. proposed a score to identify the risk of developing CR-KP infection in rectal carriers. Four criteria are analyzed: admission to ICU, invasive abdominal procedures, chemotherapy or radiation therapy and colonization at sites besides stool (25).

A rapid diagnostic method for identification of pathogens and susceptibility profile is not always available. To optimize empiric therapy for gram-negative bloodstream infections in children, doctors at Children's Hospital of Chicago were presented with a “whole susceptibility profile” of each agent when it was identified in a blood culture. This “whole susceptibility profile” was the antibiogram of all infections caused by the same pathogen in the last year, regardless the site of identification. During the study period, 132 cases of gram-negative bloodstream infections were identified, with only 10 mismatches between the “whole susceptibility profile” and the final antibiogram (26).

### Timing of Antibiotic Prescription

Two studies support the recommendation of antibiotic prescription within the first hour of sepsis identification (4, 12). Most patients in both studies had severe disease, with 69% (4) and 79% (12) with septic shock, respectively. For patients with sepsis without shock, antibiotic administration within 3 h of the moment of sepsis recognition seems to be equally effective. This information allows clinicians to reevaluate patients that are stable, trying to differentiate between viral and bacterial infection prior to prescribing antibiotics (27).

The studies on antimicrobial timing measure the time to antibiotic administration differently. Some consider time since recognition of sepsis; others count from the first altered vital sign or the moment the patient presents to the healthcare provider. Therefore, results must be interpreted with caution and are not always comparable.

### Antimicrobial Administration

Obtaining cultures prior to antibiotic administration is of paramount importance (14, 28). Blood or other relevant corporal fluids (urine, pus, cerebrospinal fluid) should be cultured according to clinical presentation of the patient. However, clinical instability and difficulty to obtain cultures cannot delay antibiotic treatment in septic patients (14).

Blood cultures in children are a specific topic of concern, since adequate technic of obtaining the samples can be difficult in this age group. Blood culture bottles for adults require 10 ml of blood, and usually are collected in pairs (aerobic and anaerobic). Specific bottles for pediatric patients require 1–3 ml of blood and are sent only to aerobic culture. Data shows that the smaller the volume of blood, the higher the chance of growing contaminant agents instead of the real noncontaminant pathogen of the infection. Time to detection also correlates with volume of blood obtained. There is no consensus on the adequate volume of blood to collect for the pediatric patient, and this must be decided according to age and weight of each patient. For patients with CVC, paired cultures are indicated. Time to positivity and number of colonies are important information to diagnose catheter-related infections (28, 29).

Antimicrobial therapy should be given intravenously or *via* intraosseous access. Intramuscular absorption of drugs is erratic and should be avoided in critically ill patients. The oral route is not indicated in patients with sepsis due to low intestinal perfusion and consequently low absorption (30).

Doses should be adjusted according to the patient weight in the pediatric population. Specific doses for newborns are available in the literature. During the first 48 h of therapy there is no necessity of dose adjustment according to renal function. This is because acute kidney injury can be part of initial presentation but improve during treatment, leading to subtherapeutic doses (11, 31).

The main class of antimicrobials prescribed in pediatrics are the beta lactams. Its pharmacokinetic (PK) and pharmacodynamic (PD) bactericidal mechanisms require that the time the free drug remains above the minimum inhibitory concentration (MIC) is more than 40–70% of time (30, 32). Prolonged infusion of beta lactams is a strategy to increase time above the MIC when pathogens have a higher MIC against a specific drug. Studies with meropenem prolonged infusion (3 h) vs. regular infusion (30 min) show no benefit in 30-day mortality. Also, the prolonged infusion requires more time using the same intravenous line, which can be difficult in unstable patients. This strategy has a role in multidrug-resistant infections with few therapeutic options and can be prescribed once the antibiotic has already reached its steady state. The first doses of the antibiotic must be administered in bolus (33).

A strategy to rapidly obtain therapeutic concentrations is using loading doses for antibiotics with best PK-PD parameters being area under the curve above the MIC. Vancomycin is an example of this strategy. With the global increase in MICs for *Staphylococcus aureus*, there is a higher risk of therapeutic failure with regular vancomycin doses (32). Two reviews showed better results with loading doses in adults, but in pediatric patients the benefits were not clear and the occurrence of side effects as the “red-man syndrome” was more frequent (34, 35).

### Definitive Therapy and De-escalation

Inpatients receiving antibiotics should be reevaluated every day. Culture results, susceptibility profile and other molecular tests should be reviewed, and patient improvement should also guide clinical decisions. The best drug for definitive treatment is the one with narrowest spectrum that is effective for the identified agent. Source control is another key component when treating sepsis, and the lack of appropriate surgical intervention is sometimes the cause of treatment failure (36).

In 28–89% of sepsis cases there is no identification of any bug. This can be due to administration of antibiotics prior to obtention of cultures, wrong technic when obtaining the samples, misdiagnose, infection caused by bacteria that is difficult to cultivate or viral infection. Data on outcomes of culture negative sepsis is controversial, with some studies showing worse outcomes when a pathogen is identified while others found similar morbidity and mortality (37). Coagulase-negative staphylococci (CoNS) are the most common agents identified in blood cultures, but not always the cause of infection. They can be contaminants of blood cultures. CoNS tend to be contaminants when there is only one positive culture and the cause of infection in patients with CVC and multiple positive cultures for the same CoNS (29).

De-escalation of antimicrobial therapy is easier when there is a positive culture result but can also be done in culture negative sepsis. Some strategies are discontinuing MRSA coverage in patients with negative swabs, limiting antibiotic time to 2–3 days after source control in abdominal infections and suspending anaerobe coverage in patients with a low probability of infection with these agents. Stewardship programs and new technologies to microbial identification are also promising strategies to de-escalate therapy (37).

### When to Stop Antibiotics?

The patient is improving, the pathogen was identified, and the therapy was de-escalated. The next step is defining the best timing for switching to oral therapy and when to stop treatment. Except from central nervous system infections, primary bloodstream infections and endocarditis, all infections can be treated orally when the patient is stable, can tolerate feeding and oral drugs and has clinical and laboratorial signs of improvement (38). Biomarkers such as C-reactive protein and procalcitonin are not useful for diagnosing sepsis and septic shock but can help in this intravenous to oral transition (39, 40).

For pediatric patients, some specific concerns are the palatability of drugs, availability of liquid formulations or dispersible tablets and the possibility of dose adjusting according to the patient's weight.

Traditionally, it is recommended that the patient should not stop treatment and must complete a certain defined antibiotic course (41). This duration is based on literature and clinical practice. On the other hand, long antibiotic courses are a risk factor for development of resistance, and new trends are to shorten the time of treatment and interrupting antibiotics according to clinical improvement (41). There are new guidelines suggesting shorter duration of treatment (38, 41, 42).

### Sepsis Prevention

Up to 50% of septic patients admitted to PICUs in Brazil have healthcare-associated infections (HAI). The main actions to prevent HAI are hand hygiene, adhesion to standard and transmission-based precautions, surface cleaning and disinfection and institution of bundles to prevent catheter-related infection, catheter-related urinary tract infection and ventilator associated pneumonia (2).

Widespread vaccination against *Streptococcus pneumoniae, Haemophilus influenzae* type B and *Neisseria meningitidis* had a huge impact on preventing meningitis and other invasive bacterial infections in children. The fall of vaccine coverage, due to shortage, vaccine hesitancy, anti-vaccine movement or during the Covid-19 pandemic represents a great risk of reemergence of vaccine-preventable diseases (43).

### New Methods for Pathogen Identification and Future Perspectives

Biomarkers for rapid identification of sepsis and classification of patient severity are still lacking. There is no single laboratory test than can differentiate infectious from not infectious causes for chronic diseases decompensation, or viral from bacterial infections.

Many patients will not receive timely and adequate therapy because sepsis was not recognized during initial evaluation (27). Moreover, rapid identification of the pathogen and its susceptibility profile is also a challenge. Traditional blood cultures will yield results in 5–7 days.

New techniques for bacterial and viral identification, such as Matrix Assisted Laser Desorption Ionization–Time of Flight (MALDI-TOF) and polymerase chain reaction (PCR) can decrease time for pathogen identification and susceptibility results (44, 45).

Commercial kits that use whole blood for pathogen identification are available. Those are PCR kits than can identify a certain number of bacteria, viruses, fungi, and the presence of resistance genes, such as mecA, vanA/B and blaKPC. MALDI-TOF can also be utilized with whole blood, but the rate of contamination and false-positive results is higher. Testing blood directly from the positive culture is possible using PCR, microarray, fluorescence *in situ* hybridization (FISH) or MALDI-TOF. Time to positivity is shorter with MALDI-TOF (60 min) and costs are lower, but best results are available when testing purified cultures samples, such as bacterial pellets or subcultures. Automated microbial system cards are used to perform antibiotic susceptibility tests with few discrepancies from traditional phenotypic methods (45, 46).

A study showed that time to optimal therapy was reduced from 73 to 48 h and time to pathogen identification from 55 to 29 h after implementation MALDI-TOF and PCR. There was no impact in mortality, but a reduction was noted on antibiotic prescription for patients with blood culture contaminants, vancomycin prescription for methicillin susceptible *Staphylococcus aureus* and non-penicillin or ampicillin prescription for *Streptococcus* A and B and *Enterococcus faecalis* (44).

Elevated costs are still an important barrier to implement these new technologies in low- and middle-income countries.

## Discussion

In the 21st century, sepsis remains a major cause of morbidity and mortality in childhood and adolescence. Prevention and timely and adequate therapy are major goals to improve clinical results. Antibiotics are mandatory to treat sepsis, but indiscriminate use and resistance development can lead to higher medical costs, drug side effects and increased mortality.

The choice of empiric therapy must be assertive, and take into consideration the site of infection, local epidemiology, severity of the disease, host comorbidities and recent exposure to antibiotics. Performing blood and other site cultures is fundamental for pathogen identification and will help defining the best drug, de-escalation, and duration of treatment.

Sepsis prevention is the best strategy to reduce mortality. For community-acquired sepsis, the main intervention is increasing vaccine coverage. For HAI, preventing sepsis means reducing risk of contamination by using bundles, stimulating adequate hand-hygiene and surface cleaning, as well as following isolation precautions. Education of patients and families for sepsis awareness can help healthcare professionals to recognize and timely treat sepsis.

**Figure 1:**
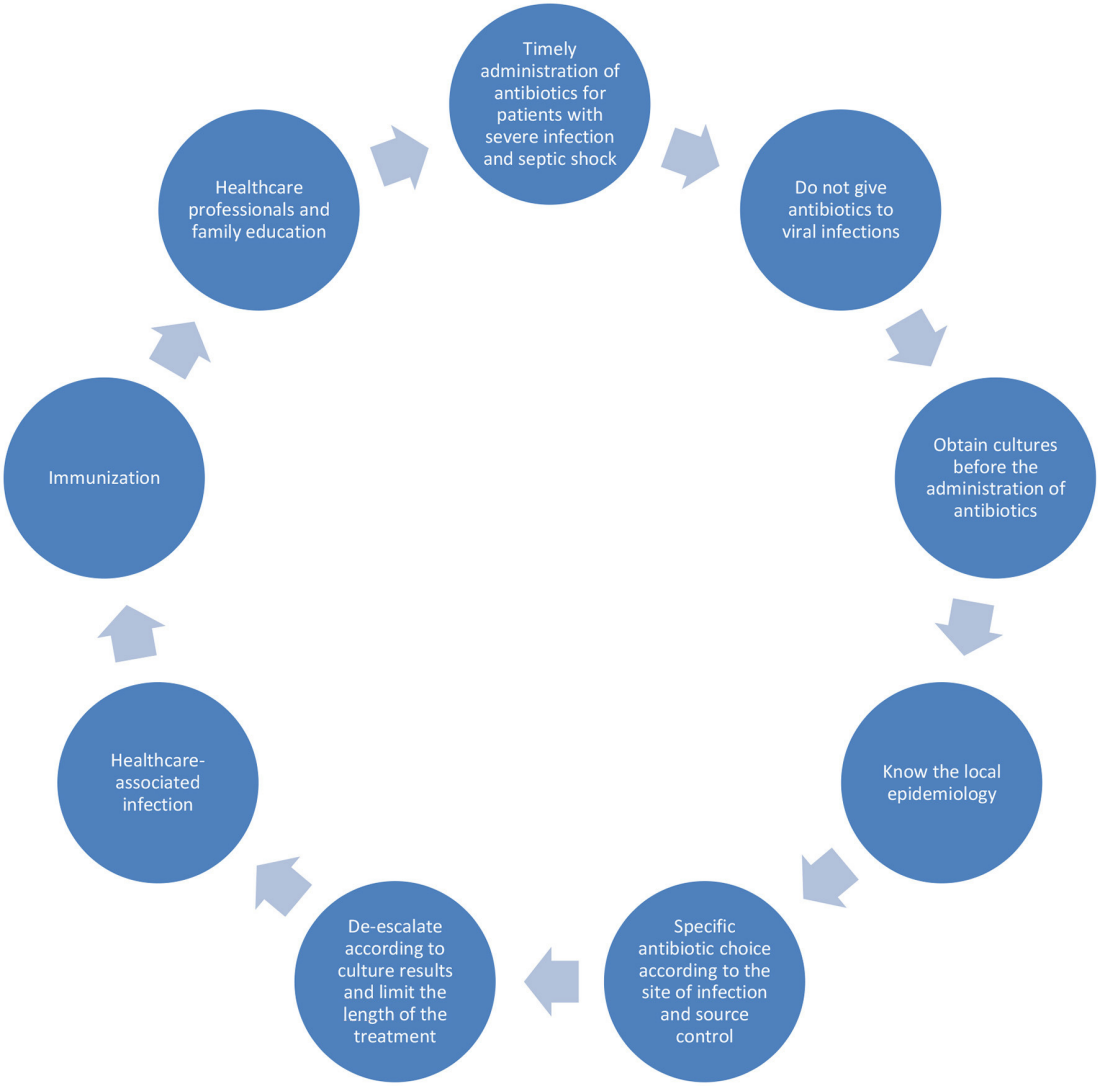
Strategies to improve outcomes in pediatric sepsis.

Future Perspectives for the treatment of sepsis are rapid diagnostic methods for pathogen identification and antibiotic susceptibility tests. Research gaps in pediatric sepsis comprise the difficulty of developing of new drugs for this age group, the small number of patients in each study and comparing methodologies in different studies, the lack of resources destinated to research in low- and middle-income countries and the difficulty in obtaining and communicating local epidemiology data (Figure 1).

## Author Contributions

LM and VB: substantial contributions to the conception and design of the work, drafting the work, and revising it critically for important intellectual content. All authors contributed to the article and approved the submitted version.

## Conflict of Interest

The authors declare that the research was conducted in the absence of any commercial or financial relationships that could be construed as a potential conflict of interest. The handling editor declared a shared affiliation with one of the author VB at time of review.

## Publisher's Note

All claims expressed in this article are solely those of the authors and do not necessarily represent those of their affiliated organizations, or those of the publisher, the editors and the reviewers. Any product that may be evaluated in this article, or claim that may be made by its manufacturer, is not guaranteed or endorsed by the publisher.
